# Intravascular papillary endothelial hyperplasia: histomorphological and immunohistochemical features

**DOI:** 10.1186/1746-1596-8-167

**Published:** 2013-10-14

**Authors:** Noyan Can Akdur, Melahat Donmez, Serap Gozel, Huseyin Ustun, Sema Hucumenoglu

**Affiliations:** 1 Pathology Department, Ministry of Health, Bursa Şevket Yılmaz Training and Research Hospital, Bursa, Turkey; 2 Pathology Department, Ministry of Health, Ankara Training and Research Hospital, Ankara, Turkey; 3 Pathology Department, Kafkas University, Faculty of Medicine, Kars, Turkey

## Abstract

**Background:**

Intravascular papillary endothelial hyperplasia (IPEH) is a benign intravascular process with features mimicking other benign and malignant vascular proliferations. IPEH lesions predominate in the head-neck region and the extremities. The characteristic histomorphological feature of IPEH is a papillary structure covered with hyperplastic endothelial cells within the vascular lumen. It is critical that this clinically benign lesion should not be mistaken for well-differentiated vascular tumors. In addition to the characteristic histological features, other useful diagnostic features included the intra-luminal location of the lesion, an intimate association with the organizing thrombus, the absence of necrosis, cellular pleomorphism, and mitotic activity. In addition, immunohistochemistry may indicate the vascular origin and proliferative index. In this study, we evaluated histomorphological and immunohistochemical findings (CD31, CD34, FVIII, type IV collagen, SMA, MSA, CD105, and Ki-67 staining) of ten IPEH cases.

**Methods:**

Ten IPEH cases were re-examined for a panel of histomorphological and immunohistochemical features. CD31, CD34, FVIII, Type IV collagen, SMA and MSA antibodies utilized for immunohistochemical analysis.

The histomorphological and immunohistochemical findings were evaluated by two independent pathologists using light microscopy.

**Results:**

All ten cases involved intraluminal lesions with characteristic features of IPEH. All ten cases (100%) were stained positive for CD31 and CD34. The degree of staining with FVIII, type IV collagen, SMA, and MSA was variable.

**Conclusion:**

In this series of specimens, CD31 and CD34 were the most sensitive markers indicating the vascular origin of the lesion. Staining for the other vascular markers (FVIII, type IV collagen, SMA and MSA) was variable. Different maturation degrees of lesions may account for the variation in immunohistochemical staining. Few previous investigations evaluated a wide range of antigen panels in IPEH sections. In our opinion, the evaluation of immune markers in a larger sample set will reveal new features in the maturity and developmental pathogenesis of vascular lesions and angiogenesis. IPEH is a benign lesion, which must be differentiated from malignant tumors such as angiosarcoma and Kaposi’s sarcoma. Improved definition of IPEH lesions using immunohistochemical markers may enhance the ability to differentiate between various vascular lesions.

**Virtual slides:**

The virtual slide(s) for this article can be found here: http://www.diagnosticpathology.diagnomx.eu/vs/1381849312101856.

## Introduction

Intravascular papillary endothelial hyperplasia (IPEH) is a benign, non-neoplastic intravascular lesion. The clinical features of IPEH may mimic other benign lesions including mucocele, pyogenic granuloma, and hemangioma, as well as malignant neoplasms such as angiosarcoma and Kaposi’s sarcoma [1-7]. IPEH is alternately referred to as Masson’s tumor, intravascular angiomatosis, and intravascular vegetating hemangioendothelioma by the many investigators who have struggles to define this unique structure [2,3]. The lesion features associated with the disease was first defined by Masson in 1923 and was termed IPEH in 1976 by Clearkin and Enzinger [1,8]. IPEH lesions frequently develop in the extremities, including the head, neck, and body, but are most prominent in the digits and within blood vessels throughout the body [1,2,9]. In rare circumstances, atypical IPEH lesions have been observed in the abdominal organs or in intracranial aneurysms [9,10].

The primary histologic feature of IPEH is the formation of papillary structures lined by hyperplastic endothelial cells in the vascular lumen [1-5,11]. IPEH is closely associated with thrombus formation in many cases. Previous reports have suggested that unique variation in thrombus organization contributes to IPEH [1,3,4,12], however the molecular basis for the development of IPEH in thrombus tissue has not been determined.

The differentiation of benign biological IPEH lesions from angiosarcoma is critical. Benign IPEH lesions are completely cured by local excision, while angiosarcoma is a malignant tumor that is capable of metastasis and may not be fully eradicated by localized surgical removal [2,4]. Several additional criteria are important in differentiating IPEH lesions from malignant angiosarcoma including intraluminal lesion origin, minimal necrosis, close association with organized thrombus, and lack of pleomorphic and mitotic activity in cells [1-4]. Although IPEH is mostly an intravascular lesion, extravascular hematoma organization features may also be present [13]. The demonstration of vascular origin and proliferative index by immunohistochemistry may contribute to the accurate differential diagnosis of IPEH [4].

In the present study, we investigate the morphological and immunohistochemical staining characteristics (CD31, C34, FVIII, Type 4 collagen, SMA, MSA, CD105 and KI-67) of 10 IPEH cases. Previous studies have used more limited immunological panels in similar analyses. In our opinion, the evaluation of immune markers in a wider series will reveal the new insights regarding the developmental stages of vascular lesions and angiogenesis. IPEH is a benign lesion that must be accurately differentiated from malignant angiosarcoma. Improved definition of IPEH lesions using immunohistochemical markers may enhance the ability to differentiate between various vascular lesions.

## Materials and methods

Hematoxylin-eosin stained sections of ten IPEH cases presenting at our hospital were re-examined for histomorphological features using light microscopy.

Four micron thick sections were prepared from paraffin blocks and mounted on to poly-L-lysine coated slides. Sections were deparaffinized overnight and then heated at 60°C to 37°C in an oven. The sections were subsequently subjected to xylene for 5 minutes three times, grade alcohol, and distilled water 3 times for 5 minutes. Antigen retrieval was performed using 10 mM citrate buffer at pH 6.0. The sections were boiled in distilled water using the equivalent of a 750-watt microwave oven for 5 minutes at a temperature interval of 20 minutes. After 20 min at room temperature, the samples were washed twice in phosphate buffer saline PBS. Sections were dampened in a drying environment of 25°C by dropping 3% hydrogen peroxide on to the sections for 15 minutes to block endogenous peroxidase activity. The primary antibody was applied without additional washing.

The following primary antibody preparations were added to each of the sections: 1/100 diluted CD31, clone: PECAM-1 (Genemed, USA); 1/100 diluted CD34, clone: BI-3C5 (Invitrogen); ready-to-use FVIII clone: 86PECAM-1 (Labvision, Fremont, CA); ready-to-use type 4 collagen clone: COL-94 (Biogenex, CA), 1/50 diluted SMA clone: ASM-1 (Leica, UK); 1/100 diluted MSA, clone: HHF35 (Genemed, USA); ready-to-use CD105, clone: SN6h (Neomarkers, Fremont, CA); 1/100 diluted Ki-76, clone: MM1 (Leica, UK). All sections were incubated with primary antibody for 1 hour. The sections were washed twice in PBS for 3 minutes. After 15 minutes secondary antibody incubation, with the sections were washed with PBS before the application of streptavidin for 15 minutes. With the sections were again washed with PBS for 10 minutes before the application of AEC chromogen. The sections were again washed with distilled water and counter-stained with hematoxylin Mayer and covered with water-based medium.

In each case, the vessels of the surrounding tissue were used as internal controls for CD31, C34, FVIII, Type 4 collagen, SMA and MSA; tonsil tissue was used as a control for CD105 and Ki-67.

## Results

**Figure 1:**
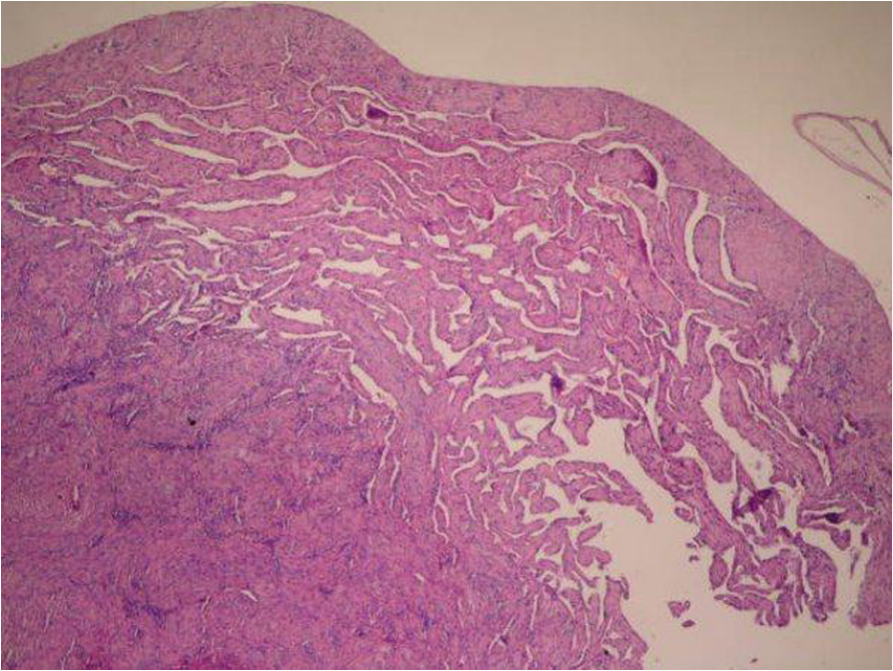
Intravascular Papillar Endothelial with organized thrombus in the lumen of a blood vessel (H&E × 40).

**Figure 2:**
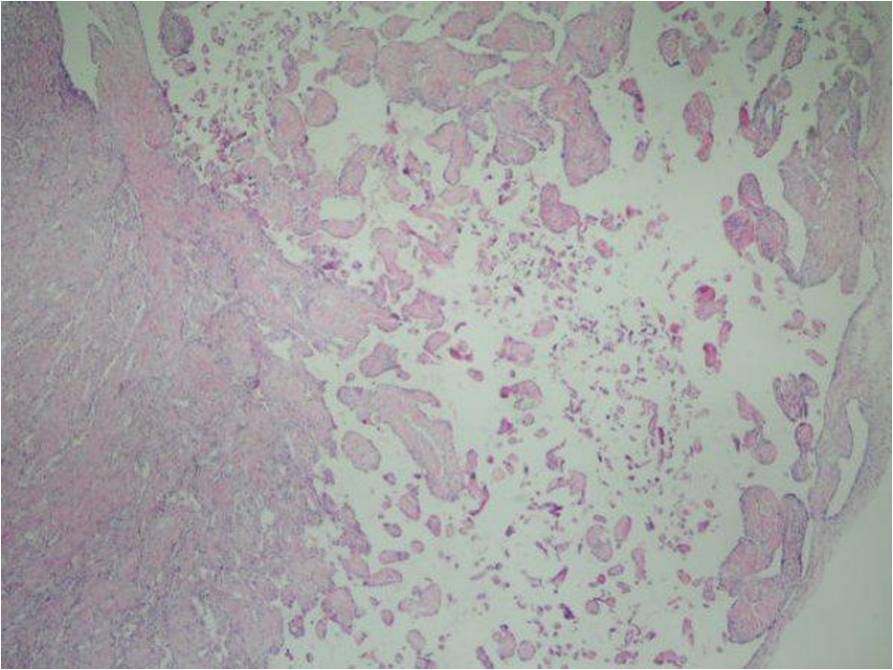
Papillary endothelial structures (H&E × 200).

**Figure 3:**
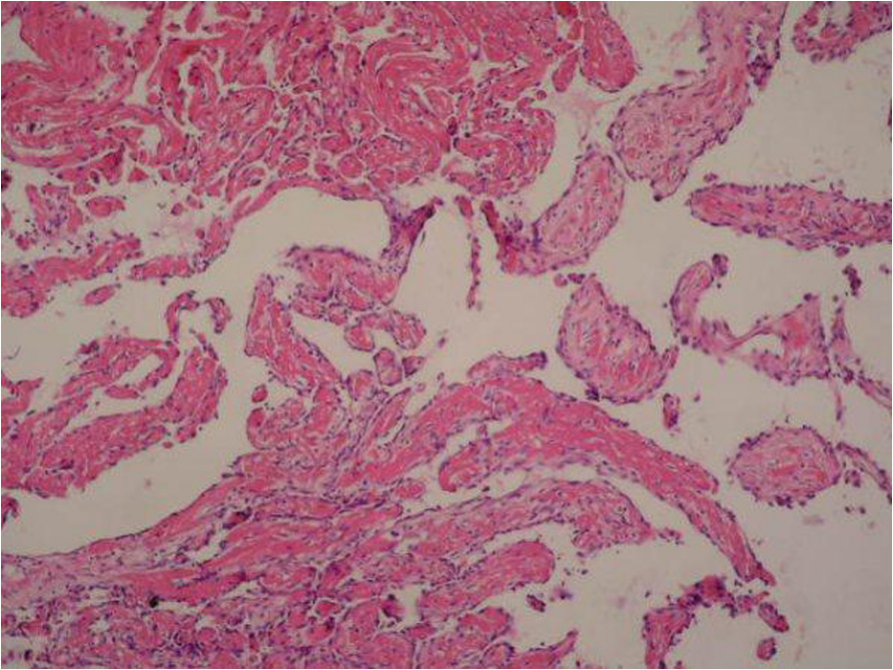
IPEH; papillary structures in the vessel (H&E × 40).

**Figure 4:**
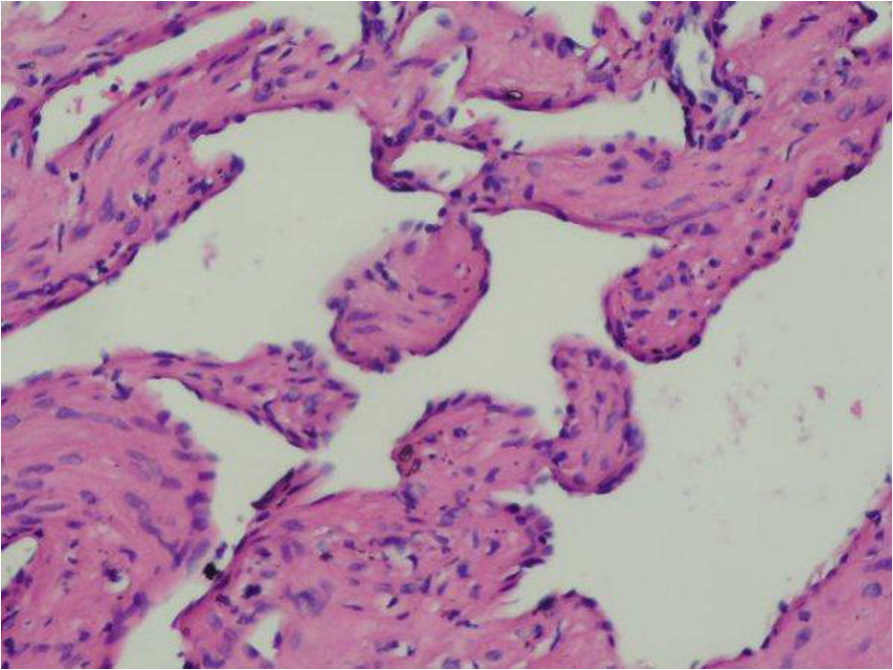
Fibrous stroma and the surrounding flattened endothelial cells in a single row, (H&E × 400).

The clinical characteristics of the IPEH cases including age, sex, localization, presentation and size is shown in detail in Table 1. The age of the patients ranged between 23 and 71, with a mean age of 40. Three of the patients were male (30%), and seven patients were female (70%). Five of the cases (50%) involved upper extremity localization, while five cases were localized in the head-neck region (50%). The largest lesion was 1.5 cm in diameter, while the smallest lesion had a diameter of 0.6 cm. The average diameter of all lesions was 0.88 cm. All cases were of confirmed intravascular localization and demonstrated specific IPEH histomophological characteristics (Figures 1 and 2). Six cases were of associated with an organized thrombus (Figure 3). The presence of papillary structures lined by a single row of endothelial cells (Figure 4), absence of pleomorphism, mitosis, and necrosis in vascular lumen was confirmed in all ten cases.

**Table 1:** Evaluation of IPEH cases in terms of age, sex, localization, presentation and size

**Case**	**Age**	**Gender**	**Localization**	**Presentation**	**Size (cm)**
1	35	male	scalp	mass	1,1 cm
2	33	female	Orbital region	mass	1,1 cm
3	45	female	hand	mass	1,0 cm
4	40	female	neck	mass	0,6 cm
5	51	male	hand	mass	0,7 cm
6	71	female	finger	mass	0,6 cm
7	27	female	hand	mass	1,1 cm
8	23	female	scalp	mass	0,6 cm
9	47	female	finger	mass	0,8 cm
10	28	male	scalp	mass	1,2 cm

**Figure 5:**
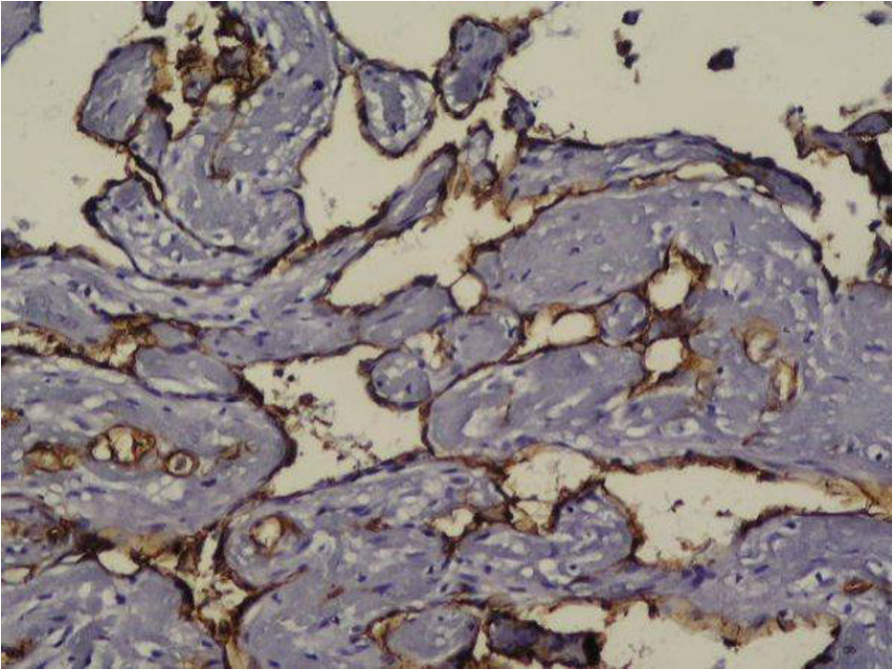
Endothelial cells stained with CD34 (×400).

**Figure 6:**
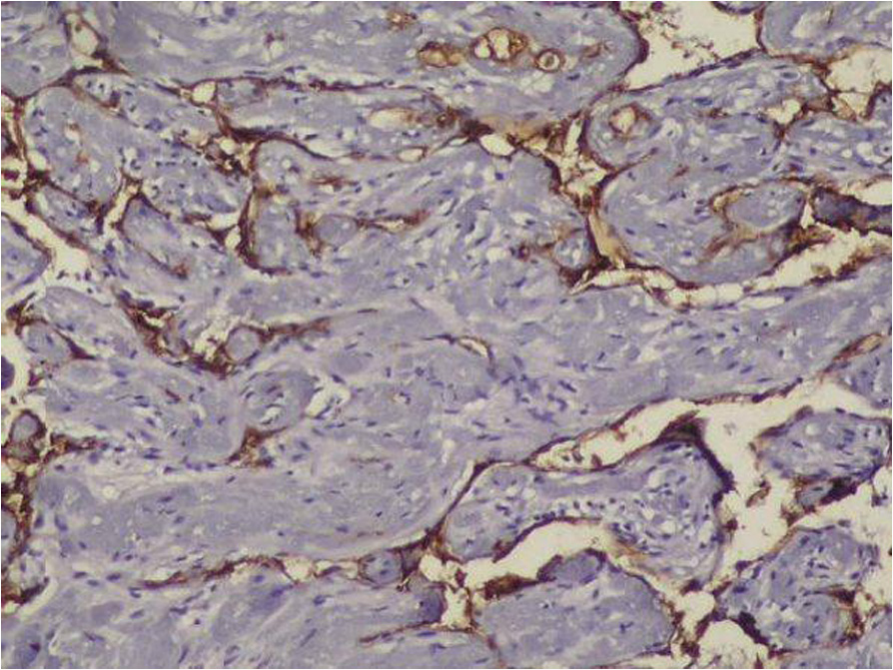
Endothelial cells stained with CD31 (×400).

The immunohistochemical staining distribution is shown in Table 2. In 5 cases (50%); CD31, CD34, FVIII, Type 4 collagen, SMA and MSA markers was observed as positive. The remaining cases demonstrated variable staining for these markers. Staining by CD31 and CD34 was present in all 10 cases (100%) (Figures 5 and 6) while staining by FVIII was observed in 6 cases (60%). Type 4 collagen staining was present in 8 cases (80%). SMA and MSA co-staining occurred in 8 cases, with only 2 cases (20%) having no reactivity for these immune markers. None of the cases stained positive for CD105. The proliferative index was less than 8% (1%-8%) for the cases examined.

**Table 2:** Immunohistochemical staining distribution

**Case**	CD31*	CD34*	FVIII**	Type IV Collagen***	SMA****	MSA****	**CD105**	**Ki67**
Case 1	+	+	+	+	+	+	-	≤1%
Case 2	+	+	+	+	+	+	-	≤1%
Case 3	+	+	-	+	+	+	-	≤2%
Case 4	+	+	+	+	+	+	-	≤8%
Case 5	+	+	-	-	-	-	-	≤5%
Case 6	+	+	+	+	+	+	-	≤7%
Case 7	+	+	-	+	+	+	-	2%
Case 8	+	+	-	-	-	-	-	3%
Case 9	+	+	+	+	+	+	-	3%
Case 10	+	+	+	+	+	+	-	1%

* They stained the immature endothelium which covers multiple small papillary structures (lining endothelial cells), and also mature well formed vessels.

** Positive staining was seen in mature lesions.

***Positive staining in the basement membrane of the endothelial vessel wall.

****Positive staining was seen only surrounding wall of mature well formed vessels.

## Discussion and conclusion

Basic histological feature of IPEH is the formation of papillary structures lined by hyperplastic endothelial cells in the vascular lumen [1-5,11]. Frequently, IPEH has a close association with thrombus. Several reports have proposed that there is a special variation in the organization procedure of thrombus [1,3,4,12], however, the basis for the development of the lesion in thrombus has not been determined. The developmental procedure of the lesion takes place in several steps [14].

Normally older trombi tend to become organized. This refers to ingrowth of endothelial cells, smooth muscle cells, and fibroblasts into the fibrin-rich thrombus. In time, capillary channels are formed, which may anastomose to create conduits from one and of the thrombus to the other, reestablishing to some extent the continuity of the original lumen. This recanalization may eventually convert the thrombus into a vascularised mass of connective tissue, which is incorporated as a subendothelial swelling of the vessel wall. With time and contraction of the mesenchimal cells, only a fibrous lump may remain to mark the original thrombus site [15].

Similar to thrombus organization and recanalization, the developmental pathogenesis of IPEH lesion formation occurs in several steps [14]. Embedment of endothelial cells within the thrombus characterizes early stage lesions. Subsequently, the proliferating endothelial cells segregate the collagenase-digested thrombus into irregular clumps from which papillary structures will develop. In the final stage, papillae combine and form anastomosing vascular structures.

Some investigators have proposed that the developing thrombus serves as a matrix for the ingrowth of papillary structures [16]. Ultrastructurally, these papillary structures closely resemble granulation tissue, suggesting a reparative origin [17]. In addition, endothelium-lining cells appear to originate in histiocytes and the exuberant endothelial proliferation involves an autocrine loop of endothelial secretions including basic fibroblast growth factor [18]. Reports of occasional cases that did not involve thrombosis in addition to reports of the presence of a lymphatic counterpart have led some authors to support Masson’s original theory that IPEH is a benign tumor marked by primary endothelial proliferation and secondary thrombus formation [19].

IPEH is a benign behavioral vascular lesion that must be accurately differentiated from malignant angiosarcoma and other vascular tumours [1-5,20]. Correct diagnosis consists of careful histomorphological examination in conjunction with IHC staining.

Monoclonal antibodies directed against CD34 and CD31 have yielded insights into the nature of vascular tumors. These antigens are not endothelial cell specific, but they are widely expressed by vascular endothelium, particularly under pathological conditions [21].

CD34 is a cell surface protein that is expressed by human hematopoietic cells of both the myeloid and lymphoid lineage, as well as endothelial cells. CD34 may regulate the early events of blood cell differentiation and modulate adhesion in both endothelial cells and hematopoietic progenitor cells [22].

CD31 is a trans-membrane glycoprotein expressed by platelets, monocytes, granulocytes, B-cells, certain subsets of leukocytes, and endothelial cells [22].

We observed CD31 and CD34 staining in all 10 cases (100%). In our opinion both CD31 and CD34 stain IPEH endothelium with high intensity and are highly effective in establishing the vascular root of the lesion. In our study CD31 and CD34 labeled all maturity levels of lesions. They stained diffuse strongly the immature endothelium which covers multiple small papillary structures (lining endothelial cells), and also mature well formed vessels,

Factor VIII-related antigen (FVIII) is a protein that is synthesized by endothelial cells and is an excellent marker of endothelial differentiation [9].

Tosios et al. have described the presence of FVIII-related antigen in the final stages of IPEH organization [23], and the presence of FVIII-related antigen is strong evidence of IPEH.

Flope et al. observed that well differentiated capillaries strongly express factor VIII, but is not expressed in the endothelial cells lining small slit-like, sieve-like vascular spaces and spindle-shaped tumor cells. Jones et al., Mentzel et al. and Wilken et al. have reported similar observations [21,24-26].

In our study 6 cases (60%) exhibited FVIII staining, similar to the findings of Albrecht and Kahn [12]. In their study, Albrecht and Kahn presented maturity-dependent variation in FVIII staining of IPEH lesions. FVIII positive staining was seen only in mature lesions.

In addition to endothelial cells, IPEH lesions consist of basal membrane and pericytes associated with vascular proliferation and immune markers. Effective identification of these components using IHC may improve IPEH diagnosis. Multiple reports have established the use of a panel of immune markers to demonstrate the vascular root of IPEH [4,5,12,21,27]. We evaluated Type 4 collagen, SMA, and MSA staining in addition to the established endothelial-specific markers in this study, observing variation in staining intensity in many of the cases examined. This variability may be related to the stage of IPEH lesion development.

Soares et al. has described Collagen type IV staining in the basement membrane of the endothelial vessel wall, and cells surrounding the vessel wall express SMA [5].

We also examined the immune marker CD105. CD105 (endoglin) is a membrane-bound homodimer expressed in angiogenic endothelial cells that has recently been associated with tumor angiogenesis. CD105 has an important role in angiogenesis and is essential for the proliferation of endothelial cells during the active phase of angiogenesis. Endothelial cells are the principal source of CD105, however other cells types including vascular smooth muscle cells, fibroblasts, and macrophages express CD105 to a lesser extent [28]. The expression of CD105 is a prominent feature of newly formed blood vessels, but is minimally expressed in fully formed vessels. The expression of CD105 in blood vessels surrounding IPEH lesions suggests a potential role for CD015 in tumor angiogenesis. Non-neoplastic tissues with increased angiogenic activity, such as the developing embryo and during wound remodeling, can also express limited amounts of CD105 [28-33].

Soares et al. [5] found that proliferative endothelial cells are negative for CD105 in IPEH tissues, suggesting that IPEH differs from the reactive processes occurring in pyogenic granulomas in which all cells are positive for CD105 expression. IPEH tissues are unlikely to be comprised of proliferative angiogenic tissues.

We investigated whether this new endothelial marker, CD105, was not present in 10 cases of IPEH. None of the tissues evaluated demonstrated significant CD105 staining, consistent with the work done by Soares et al. [5].

Few previous studies of IPEH immunohistochemical staining have utilized a wide panel of immune markers. In our opinion, the evaluation of novel immune markers, primarily FVIII, in a wider series will enhance our understanding of vascular lesions and angiogenesis.

In conclusion, IPEH is a benign behavioral vascular lesion that must be accurately differentiated from malignant angiosarcoma through careful histomorphological examination in conjunction with immunohistochemical staining.

CD31 and CD34 are the most effective markers for identification of the vascular root, whereas FVIII, Type 4 collagen, SMA and MSA staining vary widely between individual cases.

## Competing interest

The authors declare that they have no competing interest.

## Authors’ contributions

All authors of this research paper have directly participated in the planning, execution, or analysis of this study. All authors read and approved the final version submitted.
